# Statewide implementation of the cardiac arrest registry to enhance survival in Ohio

**DOI:** 10.1016/j.resplu.2023.100528

**Published:** 2023-12-16

**Authors:** Michelle M.J. Nassal, Henry E. Wang, Justin L. Benoit, Alexander Kuhn, Jonathan R. Powell, David Keseg, James Sauto, Ashish R. Panchal

**Affiliations:** a Department of Emergency Medicine, The Ohio State University, Columbus, OH, United States; b Department of Emergency Medicine, University of Cincinnati, Cincinnati, OH, United States; c Department of Emergency Medicine, Cleveland Clinic Foundation, Cleveland, OH, United States; d American Heart Association, Dallas, TX, United States

**Keywords:** Cardiac arrest, Resuscitation, Cardiac arrest registry

## Abstract

**Objective:**

Public health surveillance is essential for improving community health. The Cardiac Arrest Registry to Enhance Survival (CARES) is a surveillance system for out-of-hospital cardiac arrest (OHCA). We describe results of the organized statewide implementation of Ohio CARES.

**Methods:**

We performed a retrospective analysis of CARES enactment in Ohio. Key elements included: establishment of statewide leadership, appointment of a dedicated coordinator, conversion to a statewide subscription, statewide dissemination of information, fundraising from internal and external stakeholders, and conduct of resuscitation academies. We identified all adult (≥18 years) OHCA reported in the registry during 2013–2020. We evaluated OHCA characteristics before (2013–2015) and after (2016–2019) statewide implementation using chi-square test. We evaluated trends in OHCA outcomes using the Cochran-Armitage test of trend.

**Results:**

Statewide CARES promotion increased participation from 2 (urban) to 136 (129 urban, 7 rural) EMS agencies. Covered population increased from 1.2 M (10% of state) to 4.8 M (41% of state). After statewide implementation, OHCA populations increased male (58.1% vs 60.8%, p < 0.01), white (50.1% vs 63.7%, p < 0.01), bystander witnessed (26.9% vs 32.9%, p < 0.01) OHCAs. Bystander CPR (34.7% vs 33.2%, p = 0.22), bystander AED (13.5% vs 12.3%, p = 0.55) and initial rhythm (shockable 18.0% vs 18.3%, p = 0.32) did not change. From 2013 to 2019 there were temporal increases in ROSC (29.7% to 31.9%, p-trend = 0.028), survival (7.4% to 12.3%, p-trend < 0.001) and survival with good neurologic outcome (5.6% to 8.6%, p-trend = 0.047).

**Conclusion:**

The organized statewide implementation of CARES in Ohio was associated with marked increases in community uptake and concurrent observed improvements in patient outcomes. These results highlight key lessons for community-wide fostering of OHCA surveillance.

## Introduction

Out-of-hospital cardiac arrest (OHCA) is a leading public health crisis affecting over 500,000 individuals annually in the United States.^1^, ^2^ Evidence-based guidelines underscore the importance of community-based approaches to improving OHCA care and outcomes, including strengthening early recognition, bystander interventions, accelerating Emergency Medical Services (EMS) care, and protocolizing post-arrest care.^3^ Despite these guidelines, profound regional variability in OHCA care and outcomes exist, with survival rates varying 10-fold across the United States.^3^, ^4^, ^5^

An important impediment to closing OHCA performance gaps is the availability of structured regional data to guide system improvements and motivate improvements in clinical care.^5^, ^6^ The Cardiac Arrest Registry to Enhance Survival (CARES) is a national registry developed to improve EMS and community OHCA care.^7^ Since its inception in 2004, CARES has provided essential lessons on how to improve OHCA outcomes, such as the importance of bystander CPR on neurologically intact survival.^8^ While originally implemented by individual EMS agencies in the United States, in 2011 CARES applied strategies to implement the registry at the state level, with the goal of more broadly influencing regional OHCA care and outcomes^9^. Limited data to date describe the community uptake of this statewide approach to CARES and the resulting longitudinal influences on OHCA care and outcomes. In 2016 the State of Ohio transitioned from individual EMS agency participation, adopting a statewide implementation of CARES. We describe the process and results of the organized statewide fostering of CARES in Ohio.

## Methods

### Study setting and design

We performed a retrospective analysis of statewide CARES development in Ohio. This study was approved by the Ohio CARES Data Sharing committee and The Ohio State University Office of Responsible Research Practices. Since its inception, CARES has been deemed a public health initiative by the Centers for Disease Control and Prevention (CDC).

### Data set

CARES is a national OHCA registry managed by Emory University and the CDC in Atlanta, Georgia that prospectively collects data on non-traumatic OHCA. CARES obtains data through three resources: 9-1-1 dispatch centers, EMS providers, and receiving hospitals. EMS agencies and hospitals submit predefined data elements describing OHCA characteristics, interventions, and outcomes to the web-based registry. CARES has strict participation requirements of > 99% data entry and accuracy to be included in the dataset. This is required of individual EMS agencies and statewide subscriptions.^10^ Data entry and accuracy is confirmed by the state coordinator in Ohio. Data is entered by each site and the state coordinator reviews and audits for data accuracy. National CARES provides each agency and hospital with their outcomes in comparison to state and national averages annually for internal benchmarking or quality improvement goals.

### Intervention – Implementation of CARES

Prior to 2016, Ohio EMS agencies individually subscribed to CARES. In 2016, statewide stakeholders (including EMS agencies, state governmental representatives, local EMS leadership, and hospital system leadership) established an organized effort to implement CARES throughout the State of Ohio. The task force established a state-based subscription to national CARES and secured funding from local hospitals, academic medical centers, and the state EMS office.

Key elements of the Ohio CARES statewide enactment included: (1) establishment of a board of directors; (2) definition of program mission, vision, goals and objectives; (3) establishment of a legal organization capable of receiving and processing funding (4) hiring a dedicated statewide paramedic coordinator; (5) conversion from an individual EMS agency to a statewide CARES registry subscription; (6) statewide dissemination of information through EMS agencies and website development; (7) conduct of Resuscitation Academies through partnerships with the HeartRescue Project and the American Heart Association to teach EMS agencies how to leverage CARES data; (8) fundraising efforts via local hospitals, academic medical centers, non-profit organizations, and EMS agencies; and (9) active solicitation and on-boarding of new EMS agencies into the registry. The Statewide paramedic coordinator is of most importance as they onboard new participating EMS agencies, confirm data entry accuracy, coordinate board of directors goals and resuscitation academies.

### Study population

For the present analysis, we included all adult (≥18 years) non-traumatic OHCA reported from January 1st, 2013, to December 31st, 2020. CARES only includes OHCA with resuscitation efforts, defined as EMS-performed CPR, and/or any defibrillation, including bystander automated external defibrillator (AED) use.^7^, ^11^

### Measures

To evaluate the uptake of CARES participation in the state and its impact, we determined the total number of potential EMS agencies eligible to participate in Ohio CARES and the population covered by the respective EMS agencies. Potential EMS reporting agencies were determined by identifying all 911-responding licensed EMS agencies, cross-referenced using the National Emergency Medical Services Information System (NEMSIS) database. Two investigators (AK and MN) performed this analysis and removed transport-only agencies (i.e. not 911-responding agencies), duplicates, and specialty agencies (e.g. non-transport amusement park agency, aeromedical agency). Population coverage by respective EMS agencies was self-reported by the EMS agency and consistent with that reported to NEMSIS. Urban vs Rural agencies were determined by EMS service area populations per the 2020 census.gov definitions.

For each OHCA, we identified age (median, interquartile range), gender (male or female), and race (white, black/African American and other), location of arrest (home, nursing home, public, healthcare facility, street, industrial place, or other), witnessed status (unwitnessed, bystander witnessed, or 911-responder witnessed) initial cardiac rhythm (shockable or non-shockable), bystander CPR performed (yes or no), bystander AED (yes or no) return of spontaneous circulation (ROSC) (yes or no), survival to hospital discharge (yes or no), and cerebral performance category score at time of hospital discharge (CPC, 1–2 or 3–4)^11^. Per national CARES definitions, bystander CPR excludes EMS-witnessed, nursing home, and healthcare facility arrests. Similarly, bystander AED use excludes OHCA occurring in homes or personal residences as well as instances of law enforcement applied AED. ROSC is defined as a sustained palpable pulse or measurable blood pressure for 20 minutes. Shockable rhythm is defined as ventricular fibrillation, pulseless ventricular tachycardia, or a shock delivered by a bystander AED, with all other cardiac rhythms or scenarios defined as non-shockable.

### Outcomes

In this evaluation, the outcomes were number of participating EMS agencies, covered population, annual number of OHCA recorded, percentage of cases with bystander cardiopulmonary resuscitation (CPR), percentage of cases with AED use, ROSC, survival to hospital discharge, and good neurological outcome (CPC 1 or 2).

### Analysis

We analyzed data using descriptive statistics. We used geospatial mapping to depict the geographic distribution of participating EMS agencies and covered population. We compared OHCA characteristics before (2013–2015) versus after (2016–2019) statewide implementation of CARES using chi-square test. We evaluated ROSC, survival, and CPC change over time using the Cochran-Armitage test of trend. We excluded 2020 from the analysis due to the recognized impact of the COVID-19 pandemic upon OHCA incidence and outcomes.^12^ We performed all analyses using STATA IC version 17 (StataCorp LP, College Station, TX), and ArcGIS (Environmental Systems Research Institute, Redlands, CA).

## Results

During the study period, there were 15,388 adult OHCA reported to Ohio CARES with an average OHCA prevalence of 84.5 per 100,000. The median age was 60, with the majority male (60.1%) and white (60.4%) (Table 1). Similar to national data, arrests primarily presented in a home residence (70.1%), were unwitnessed (54.4%) and had an initial non-shockable rhythm (81.7%). Overall rates of ROSC, survival and neurologic recovery were similar to national averages^2^. (Table 1) Comparing before and after statewide implementation, we did observe differences in OHCA patient characteristics summarized in Appendix 1. Primarily, patients after statewide implementation were higher proportion of males (60.8%), white race (63.7%) that was bystander witnessed (32.9%).

**Table 1:** Demographics of out-of-hospital cardiac arrests in the Ohio CARES registry, 2013–2020. Bystander cardiopulmonary resuscitation (CPR), bystander automatic external defibrillator (AED), return of spontaneous circulation (ROSC), cerebral performance categories (CPC) score. *Bystander CPR and AED total removed EMS witnessed arrests, nursing home arrests and Healthcare Facilities. Bystander AED total also excluded Home/Residence arrests and Law Enforcement First Responder placed AEDs.

**OHCA Characteristics**	**Total n = 15388**
Age, median (IQR)	60 (50–73)
Male, n(%)	9,253 (60.1%)
Race n(%)	
White	9,287 (60.4%)
Black/African-American	4,513 (29.3%)
Other	1,587 (10.3%)
missing	1 (0.0%)
Location of Arrest n(%)	
Home/Residence	10,787 (70.1%)
Nursing Home	1,996 (13.0%)
Public/Commercial Building	951 (6.2%)
Healthcare Facility	720 (4.7%)
Street/Highway	708 (4.6%)
Industrial Place	72 (0.5%)
Other	26 (0.2%)
Transport Center	7 (0.1%)
Witnessed Status n(%)	
Unwitnessed	8,354 (54.3%)
Bystander Witnessed	4,831 (31.4%)
911 Responder Witnessed	2,203 (14.3%)
Initial rhythm, n(%)	
Shockable	2,809 (18.3%)
Non-shockable	12,578 (81.7%)
missing	1 (0.0%)
Bystander CPR (*total n = 10,678), n(%)	3577 (33.5%)
Bystander AED (*total n = 1657), n(%)	207(12.5%)
ROSC n(%)	4,581 (29.8%)
Survival to Hospital Discharge (*total n = 15,352), n(%)	1,573 (10.2%)
missing	36 (0.2%)
CPC Score n(%)	
1–2	1,144 (7.4%)
3–4	428 (2.9%)

**Fig. 1:**
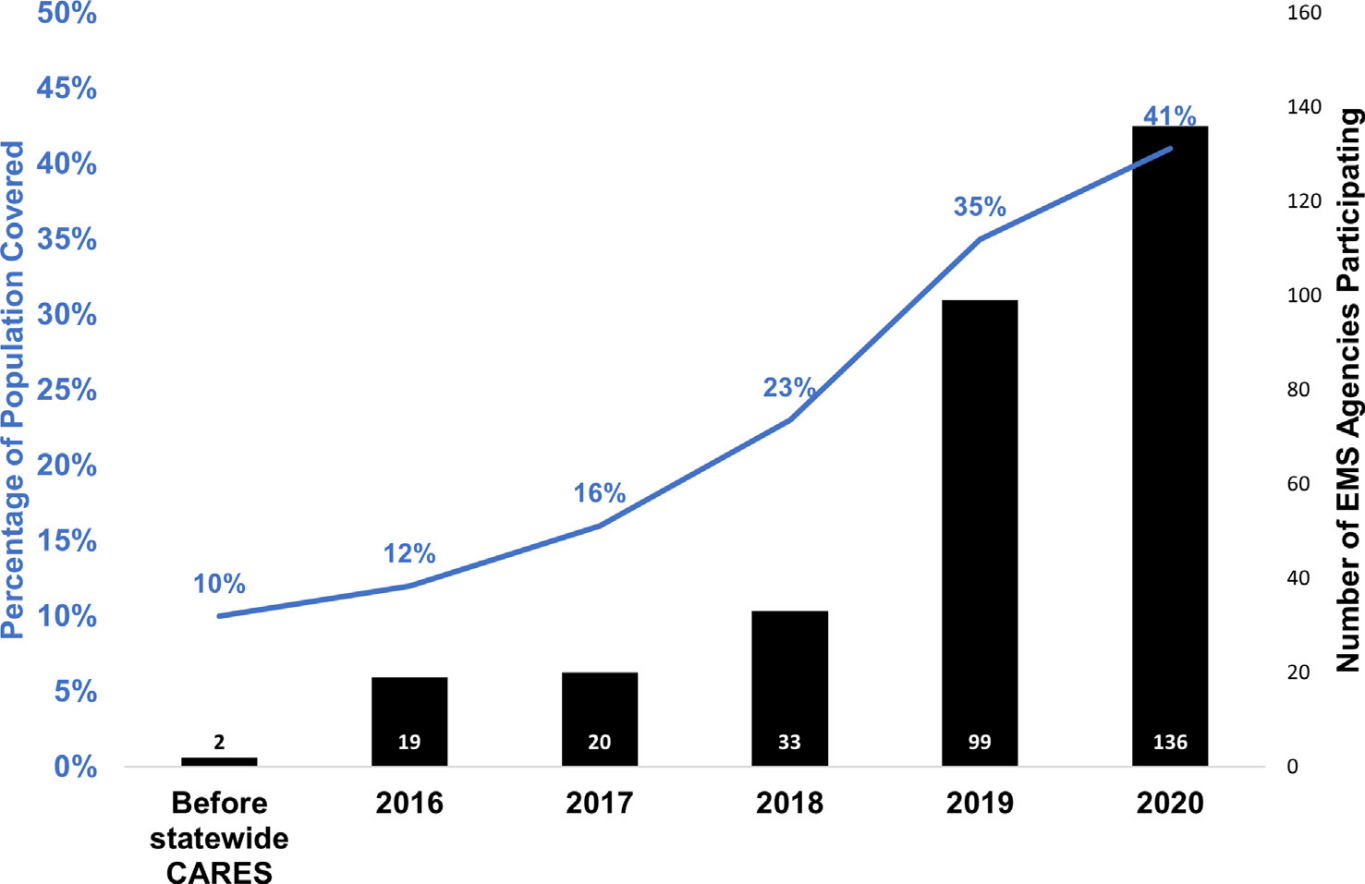
Temporal Trends in Ohio CARES EMS Agency Participation. The percentage of the population covered by Ohio CARES participating agencies is depicted by the blue line graph. The number of participating EMS agencies each year is shown by the black bar graphs.

**Fig. 2:**
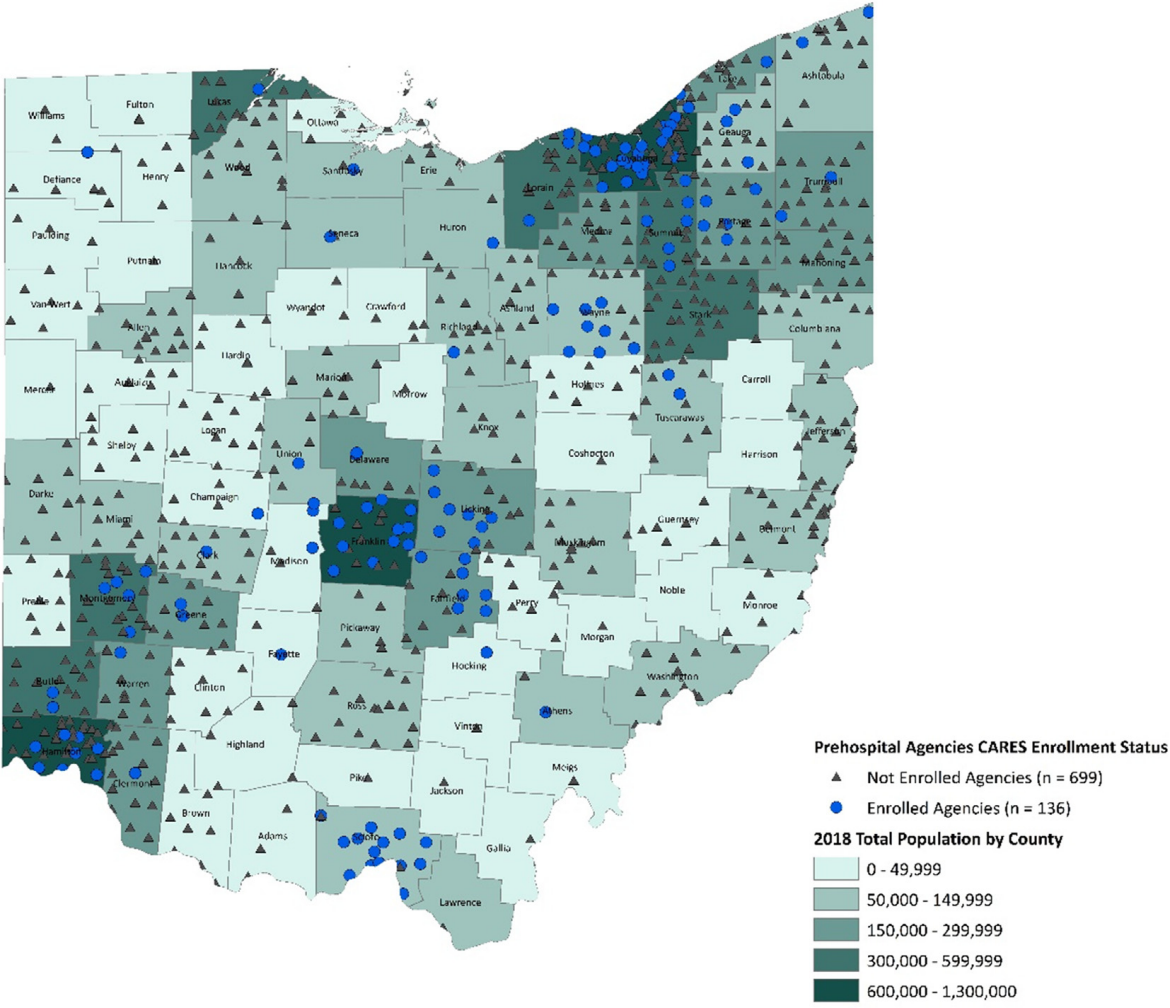
EMS agencies participating in CARES.

Prior to the launch of Ohio CARES, individual EMS agencies participating in CARES encompassed 10% of the state population. After statewide implementation in 2016, registry EMS agency participation rapidly increased (Fig. 1). During the observation period, the state population covered by Ohio CARES increased from 1.2 million to 4.8 million (41% of state). These agencies were located primarily in urban areas (Fig. 2).

**Fig. 3:**
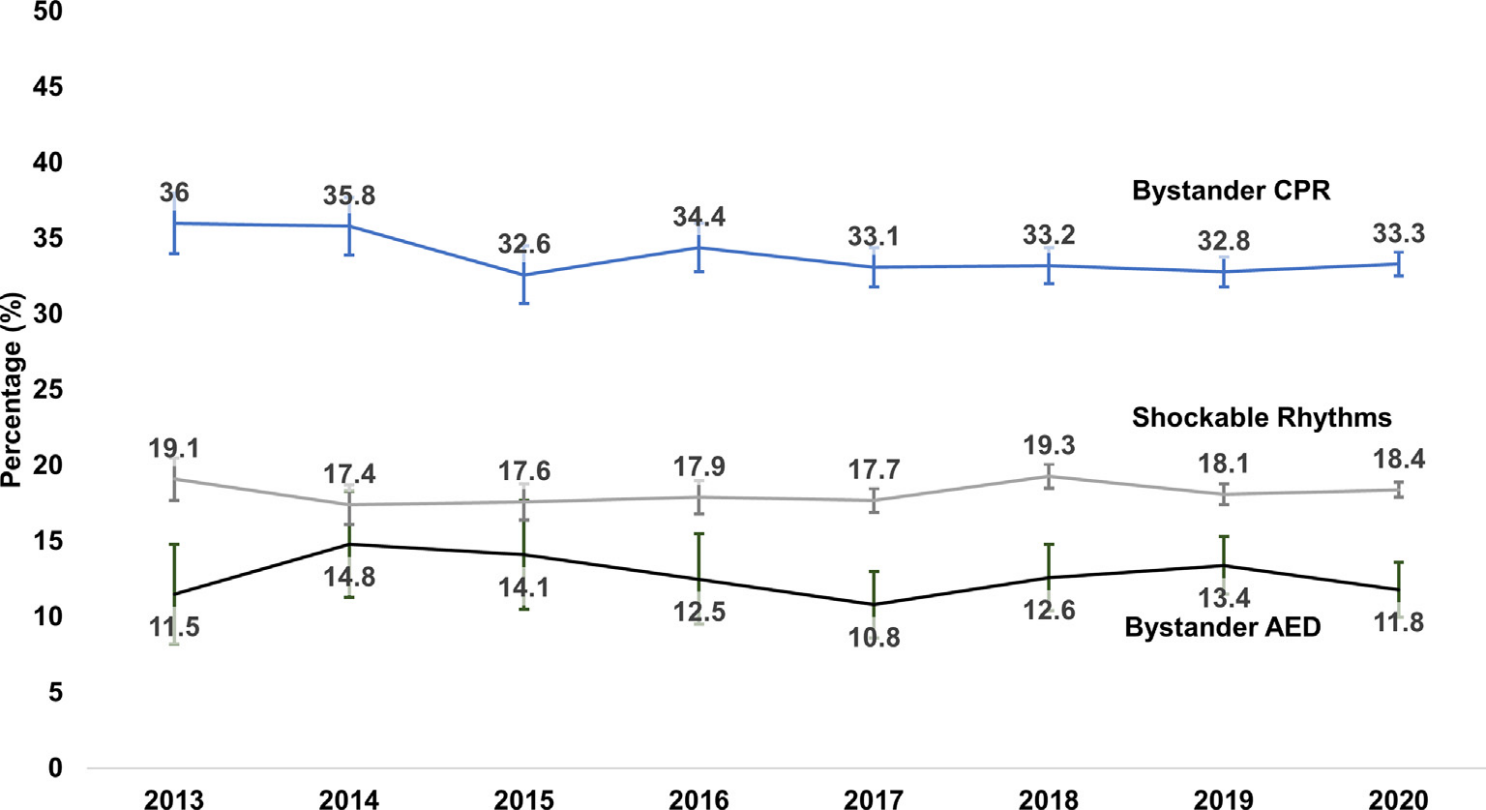
OHCA characteristics from 2013 through 2020 in Ohio CARES. Rates of bystander CPR, bystander AED and initial presenting rhythm did not change before or after implementation of a statewide based CARES registry.

Bystander CPR (before 34.7% vs after 33.2%, p = 0.22) and bystander AED use (13.5% vs 12.3%, p = 0.55) did not change between the pre- and post-implementation periods (Fig. 3). Initial presenting rhythm also did not change between pre- and post-implementation periods (shockable before 18.0% vs after 18.3%, p = 0.32).

**Fig. 4:**
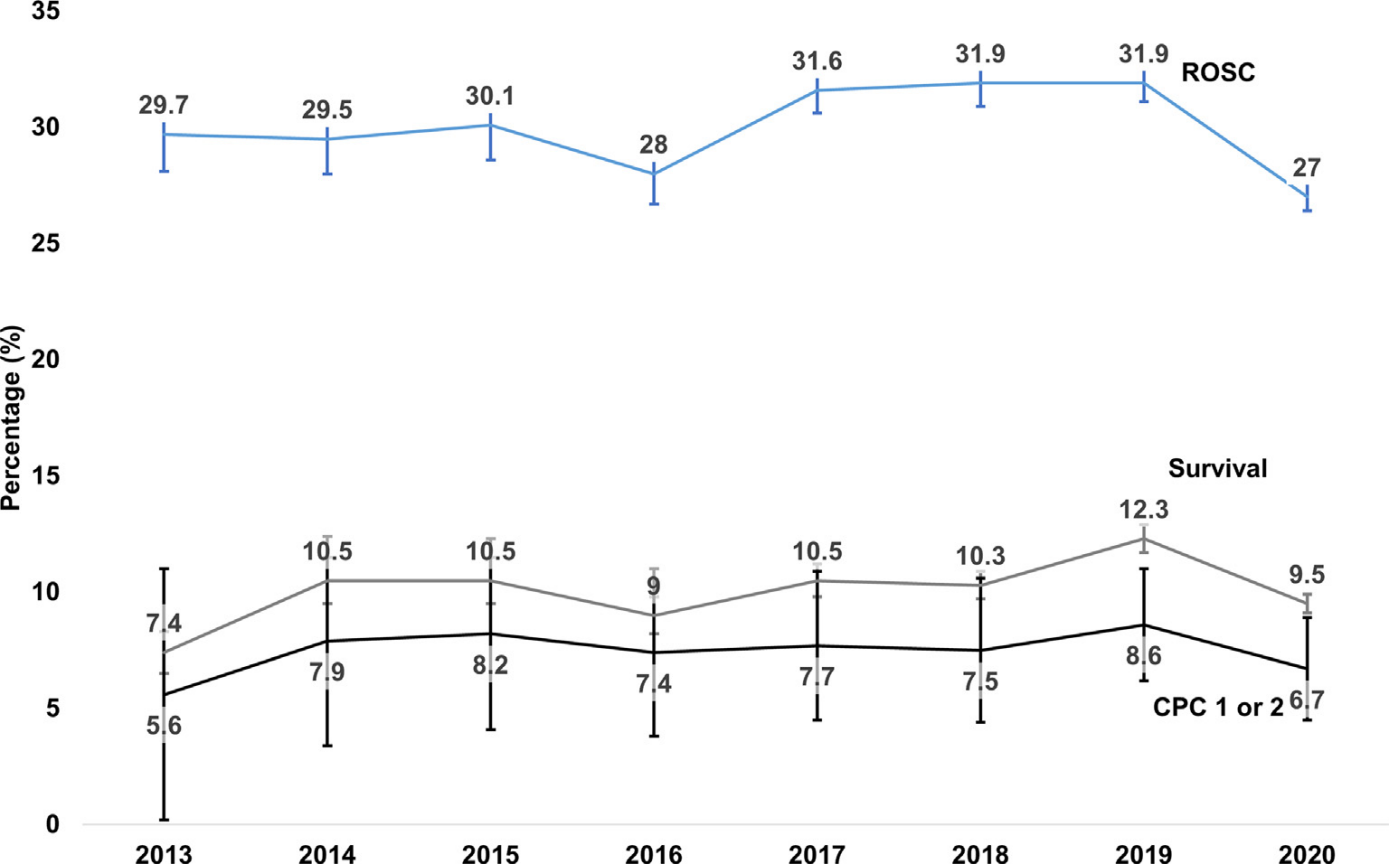
OHCA outcomes from 2013 through 2020 in Ohio CARES. Excluding 2020, rates of return of spontaneous circulation (ROSC, p-trend = 0.028), survival (p-trend < 0.001) and cerebral performance categories (CPC, p-trend = 0.047) exhibited temporal increase.

There were temporal increases in ROSC (2013: 29.7%; 2019: 31.9%, p-trend = 0.028), survival (2013: 7.4%; 2019: 12.3%, p-trend < 0.001) and neurologic outcome (2013: 5.6%; 2019: 8.6%, p-trend = 0.047) (Fig. 4).

## Discussion

The availability of community-wide data is essential for assessing the uptake and influence of any public health intervention, but the enactment of large-scale tracking and benchmarking for prehospital diseases is challenging and requires a significant commitment of infrastructure and personnel. The CARES registry transformed OHCA care in the United States, by offering a simple yet robust framework for community surveillance of OHCA. Organization of a state-based initiative accelerated community participation in CARES that had stagnated for almost a decade (2007 to 2015) with uptake temporally increasing from 10% to 41% of the state’s population within five years after statewide promotion. CARES implementation was also temporally associated with improvements in OHCA outcomes. In this evaluation, we demonstrate the power of a statewide community-based deployment of CARES and associated improvement in public health outcomes.

Similar to our findings, other states have demonstrated significant improvements in outcomes leveraging large scale registries for OHCA care innovations. Other state registries focused on increasing the public’s awareness and response to OHCA as their initial mission to improve OHCA outcomes.^13^ For example, multistate initiatives with the HeartRescue Project improved bystander CPR rates, which resulted in associated improved OHCA outcomes.^14^, ^15^ The Minnesota Resuscitation Consortium (MRC) similarly improved OHCA survival through monitoring OHCA, improving bystander CPR education and development of EMS protocols.^13^ Similarly, Arizona's statewide bystander targeted educational strategies such as chest-compression only CPR and development of designated cardiac arrest centers improved survival.^16^, ^17^.

Ohio CARES builds upon these prior efforts by coordinating statewide initiatives, focusing on training and outreach in both EMS and hospital care, in combination with wide deployment of a structured registry. One distinction of our report is the rapid implementation of state-designated goals and the associated increase in population coverage with EMS enrollment over a five-year period (Fig. 1). In comparison, other statewide CARES registries have covered 39 EMS agencies and 40.1% of the population over a six-year period.^18^ Some states have had significant success in population coverage over a shorter four-plus year period covering 70% of the population.^19^.

Further, early Ohio CARES agencies were urban with short response times, therefore we expected to observe decreased outcomes as we enrolled more EMS agencies through a statewide registry.^20^, ^21^ An important observation is that we noted secular improvements in rates of ROSC and survival with good neurologic recovery but not in rates of bystander CPR or AED use. This finding suggests that the observed trends in outcomes may be due to improvements in EMS resuscitation or in-hospital post-arrest care. However, we also noted cardiac arrest populations after statewide enrollment were more male and bystander witnessed OHCAs which may have also contributed to improved outcomes. This analysis does not determine a causal link between enhanced agency involvement and OHCA outcomes. We describe the initial development of a coordinate state registry with focus on Resuscitation Academies and state population coverage. We can support an associative improvement in outcomes with simultaneous Resuscitation Academies but it is not known if they resulted in improved outcomes. Critically reviewing our observations, it is unclear if we observe a clinically significant increase in ROSC rates (29.7% to 31.9%; however, survival (7.4% to 12.3%) with good neurologic recovery (5.6% to 8.6%) may have clinically improved over this time period. This may suggest potential changes in population characteristics or improvements in post-cardiac arrest care more than prehospital resuscitation contributed to outcome improvements.

One challenge we noted in the implementation process is that onboarding an EMS agency required substantial time over several months or years.^22^ Onboarding a new agency in Ohio CARES is estimated at 12 to 18 months and requires training facilitated by the state coordinator. Further barriers included fundraising efforts to maintain subscription costs and employment of a program coordinator to maintain data integrity (>99% data entry accuracy per agency). Other states’ CARES funding support has included grants or support from the state EMS Office. Despite these challenges, it is important to emphasize that participation of new EMS agencies and therefore more OHCA enrollments continued to increase throughout this period.

These observations support the viability and value of statewide implementation of OHCA surveillance. Unifying prehospital and in-hospital monitoring of OHCA provides benchmarks for quality improvement across the continuum of care. Identifying positive resuscitation interventions in agencies with outperforming outcomes can inform improvement strategies for underperforming agencies. Importantly, this is performed anonymously to focus solely on improvement opportunities. Furthermore, harnessing the state board to provide focused improvement strategies allows focused education through Resuscitation Academies. Current Ohio CARES board initiatives also include continued outreach and onboarding of agencies, specifically with an emphasis on rural population coverage, increasing public awareness of bystander CPR efficacy and procurement of statewide funding to establish a second coordinator for additional agency support and data accuracy.

## Limitations

We underscore that associations cannot prove causality.^23^ We recognize that other factors, such as population change as the database grows, may contribute to the temporal improvements observed in this study. Additionally, during this time period AHA published the 2015 guidelines which included recommendations for Narcan use in suspected overdose associated cardiac arrests.^24^ Large clinical trials in OHCA were also published during this prolonged time period which may have led to changes in EMS interventions.^25^, ^26^. Further, recruitment bias could have effected our observed results. Early adopting agencies may have had better outcomes than late adoption agencies; however, we would have expected a negative outcomes trend.^21^, ^22^, ^23^, ^24^, ^25^, ^26^, ^27^ Additionally, individual agencies may not have participated in each sequential year. Lastly, the COVID-19 pandemic began during this observation period which may have altered interventions such as bystander involvement.^28^, ^29^ However, we emphasize simply tracking OHCA to identify potential improvement strategies has been shown to improve outcomes through targeted interventions.^30^, ^31^, ^32^

## Conclusions

Large statewide implementation of OHCA surveillance is feasible. Organized statewide initiatives may facilitate statewide improvements in OHCA outcomes.

## CRediT authorship contribution statement

**Michelle M.J. Nassal:** . **Henry E. Wang:** Writing – review & editing, Supervision, Methodology, Conceptualization. **Justin L. Benoit:** Writing – review & editing, Resources, Conceptualization. **Alexander Kuhn:** Formal analysis, Data curation. **Jonathan Powell:** Writing – review & editing, Formal analysis, Conceptualization. **David Keseg:** Resources, Data curation, Conceptualization. **James Sauto:** Data curation, Conceptualization. **Ashish R. Panchal:** Writing – review & editing, Writing – original draft, Supervision, Resources, Methodology, Formal analysis, Data curation, Conceptualization.

## Declaration of competing interest

The authors declare that they have no known competing financial interests or personal relationships that could have appeared to influence the work reported in this paper.
