# Supplementary material for: Statewide implementation of the cardiac arrest registry to enhance survival in Ohio
Source: Resusc Plus. 2023 Dec 16;17:100528. doi: 10.1016/j.resplu.2023.100528 (PMC10765104; doi:10.1016/j.resplu.2023.100528)
Supplement: Supplementary data 1 [file mmc1.docx]

| **Patient Characteristics before and after Implementation** | **Years 2013-2016**  **n=3823** | **Years 2017-2020**  **n=11565** |
| --- | --- | --- |
| Age, median (IQR) | 61 (50-73) | 61 (50, 73) |
| Male, n(%) * | 2,220 (58.1%) | 7033(60.8%) |
| Race n(%) *  White  Black/African-American  Other  missing | 1,913 (50.1%)  1,299 (34.0%)  610 (16.0%)  1 (0.0%) | 7374 (63.7%)  3,214 (27.8%)  976 (8.4%) |
| Location of Arrest n(%) *  Home/Residence  Nursing Home  Public/Commercial Building  Healthcare Facility  Street/Highway  Industrial Place  Other  Transport Center | 2,644 (69.2%)  554 (14.5%)  225 (5.9%)  130 (3.4%)  211 (5.5%)  16 (0.4%)  43 (1.1%) | 8,143 (70.4%)  1,442 (12.6%)  726 (6.3%)  590 (5.1%)  497 (4.3%)  56 (0.5%)  8 (0.1%)  7 (0.1%) |
| Witnessed Status n(%) *  Unwitnessed  Bystander Witnessed  911 Responder Witnessed | 2,266 (59.3%)  1,030 (26.9%)  527 (13.8%) | 6,088 (52.6%)  3,081 (32.9%)  1,676 (14.5%) |

**Appendix 1**
